# Effectiveness of Methadone Maintenance Treatment in Prevention of Hepatitis C Virus Transmission among Injecting Drug Users

**DOI:** 10.5812/hepatmon.12411

**Published:** 2013-08-17

**Authors:** Seyed Moayed Alavian, Alireza Mirahmadizadeh, Mehdi Javanbakht, Ali Keshtkaran, Alireza Heidari, Atefeh Mashayekhi, Shima Salimi, Mohammad Hadian

**Affiliations:** 1 Baqiatallah Research Center for Gastrointestinal and Liver Diseases, Baqiatallah University of Medical Sciences, Tehran, IR Iran; 2 Middle East Liver Disease Center, Tehran, IR Iran; 3 HIV/AIDS Research center, Shiraz University of Medical Sciences, Shiraz, IR Iran; 4 Health Management and Economics Research Center, School of Health Management and Information Sciences, Tehran University of Medical Sciences, Tehran, IR Iran; 5 Health Management and Social Development Research Center, Golestan University of Medical Sciences, Gorgan, IR Iran; 6 Department of Health Economics, School of Health Management and Information Sciences, Iran University of Medical Sciences, Tehran, IR Iran

**Keywords:** Hepatitis C, Effectiveness, Methadone, Maintenance, Incidence, Iran

## Abstract

**Background:**

Injecting drug users (IDUs) are a major and most important risk factor for rising hepatitis C virus (HCV) prevalence in Iran.

**Objectives:**

The objective of this study was to determine the effectiveness of methadone maintenance treatment (MMT) in prevention of HCV infection transmission among IDUs.

**Patients and Methods:**

A mathematical modeling has been used to estimate number of HCV infections averted. The input parameters used in the model were collected by self-reported method from 259 IDUs before registering and one year after MMT. Nonparametric statistical tests have been used to compare risky injecting and sexual behaviors among IDUs before and after participating in MMT program. Deterministic sensitivity analyses were done to show the effects of parameters’ uncertainty on outcome.

**Results:**

Of the 259 participants, 98.4% (255) were men, the mean age ± SD was 33.1 ± 7.58 years and HCV prevalence was 50%. The studied IDUs reported lower rate of risky injecting and sexual behavior after participation in MMT program. The cumulative incidence of HCV per 100 IDUs due to sharing injection and unsafe sexual contact with MMT program were 13.84 (95% CI: 6.17 -21.51), 0.0003 (0.0001 - 0.0005) and without it 36.48 (25.84 - 47.11) and 0.0004 (0.0002-0.0006) respectively.

**Conclusions:**

The MMT program is an effective intervention to prevent HCV infection transmission, although it is essential to compare its effectiveness with other interventions before implementing it in nationwide.

## 1. Background

Chronic infection with hepatitis C virus (HCV) is increasingly recognized as a major global health problem (1, 2). Although infection with HCV is usually asymptomatic, about 70-80% of patients develop chronic infection which leads to hepatic fibrosis, cirrhosis, hepatocellular carcinoma, and death (3, 4). Numerous studies have shown that HCV infection is widespread throughout the world. It has been estimated that 2 to 3% of the global population which corresponds to about 170 million people are now infected with HCV (5). A higher seroprevalence of HCV infection has been reported among injecting drug users (IDUs) (6, 7). It has been reported that about 1% of the Iranian general population has anti-hepatitis C virus antibodies (8, 9). The range of HCV infection among Iranian’s IDUs has been estimated to be 34% to 88% (7, 10). High prevalence of HCV infection and sharing injecting equipment among IDUs constitutes an ongoing threat.

Today, IDUs are a major and perhaps the most important risk factor for rising prevalence of HCV infection in Iranian population (11). It has been estimated that between 200 to 300 thousand IDUs are now living in Iran (12). Interventions that can reduce the prevalence of high risk behaviors among IDUs are, therefore, critical components of a comprehensive hepatitis C prevention policy. Methadone maintenance treatment (MMT) is by far the most easily available treatment for addiction to heroin and other opiates. Now, extension of MMT centers and developing of their services is questionable for health policy makers and need to documentary and scientific proof.

## 2. Objectives

The aim of this study was to determine the effectiveness of MMT program in prevention of HCV infection incidence among IDUs.

## 3. Patients and Methods

### 3.1. Study Design

To estimate number of new infection, a mathematical modeling has been used. This model has been designed by Weinstein and colleagues (13) that determines the changes in drug users' possible high risk behaviors and shows the probability of transmitting infection. The input data used in the model were collected by self-reported method from 259 IDUs in seven governmental MMT centers in Shiraz, south of Iran (Table 1). Their injecting and sexual high risk behaviors before registering on MMT and one year after that were assessed. The results of high risk behaviors analysis was used to estimate total number of HCV infections averted as a measures of effectiveness.

**Table 1.** Main Characteristic of Subjects

Variable	No. (%), (Total = 259)
**Age, y**	
≤ 20	2 (0.8)
21-25	44 (17.0)
26-30	64 (24.7)
31-35	62 (23.9)
36-40	44 (17.0)
41-45	17 (6.6)
46-50	17 (6.6)
> 50	9 (3.5)
**Sex**	
Male	255 (98.4)
Female	4 (1.6)
**Marital status**	
Single	144 (55.6)
Married	68 (26.3)
Divorced	46 (17.8)
Widow	1 (0.4)
**HCV infection (n ^a^ = 138)**	
Yes	69 (50)
No	69 (50)
**HIV infection (n ^a^ = 144)**	
Yes	60 (41.7)
No	84 (58.3)
**HCV/HIV co-infection (n ^a^ = 138)**	49 (35.5)

^a^Among all studied IDUs 138 and 144 persons had HCV and HIV certified clinical test respectively

### 3.2. Estimation of Number of HCV Infections Averted

The probability of getting infection by investigated injection drug users, (A) through shared injection with other IDUs, (B) is represented by the following equation:

P_BA_ =1-[PB [(1 - ROT) ^ni/2^] + (1- PB)] ^m^

In which PB is HCV prevalence among IDUs, ROT is the rate of transmission of HCV, ni is average number of shared injections per week and m is the average number of injecting partners in each session. We assumed that the position of investigated person changes randomly in every injection session, and totally half of them are at risk of infection therefore, all injections were divided by 2 (ni/2).

We added all these possibilities and calculated the number of those likely to become infected. After determining the probability of infection, we multiplied it by the number of injecting partners and then multiplied it by the probability of their being negative, so the number of infected people by each individual was calculated. Finally, we subtracted the new estimated HCV infections before and after their arrival to MMT centers.

The probability of getting infection through sexual contact is represented by the following equation:

P_BA_ = 1-[PB [1 - ROT_BA_ (1 - f.e)] ^ns^+ (1-PB)] ^m^

In which PB is HCV prevalence among sexually high risk groups, ROT is the rate of HCV transmission with sexual contacts, ns is number of sexual acts with each partner, f is the proportion of sexual encounters in which condoms are used, e is efficacy of condom to prevent virus transmission and m is average number of sexual partners. We assumed that HCV transmission rate through shared injection and sexual contact is (0.84 – 10%) (14-16) and (7 * 10-8 - 1 * 10-^6^) (17, 18) respectively. We also assumed that condom efficacy to prevent virus transmission is (35 – 95%) (19, 20). Finally we assumed that HCV prevalence among Iranian IDUs and sex workers is (34 – 68%) (7, 10, 21) and (7-15%) (22, 23) respectively.

### 3.3. Statistical and Sensitivity Analysis

The continuous variables were expressed as mean ± standard deviation. After checking data for normality the Wilcoxon signed ranks test were used to compare means. All statistical tests were two-sided and the significance level was set at 0.05. Confidence intervals were calculated using the bias-corrected accelerated (BCA) percentile bootstrapping method. We conducted a deterministic one-way sensitivity analysis to determine the strength of the results. In which each parameter was changed in a sequence to the upper and lower limits at defined range while the other variables were held constant.

### 3.4. Ethics Statement

Before enrolment, the subjects received detailed written and verbal information regarding the aims and protocol of the study and signed informed consent. The study has been approved by the Ethics Committee of the Shiraz University of Medical Sciences.

## 4. Results

Main characteristics of the subjects are summarized in table 1. Of the 259 participants, 98.4% (255) were men and the mean age ± SD was 33.1 ± 7.58 years. Among all studied IDUs 138 people had HCV certified clinical test, which showed that HCV prevalence was 50%. Our results revealed that the subjects reported higher average number of injection per week (21.28 ± 15.11 vs. 7.74 ± 7.58), sharing injection per week (3.10 ± 5.42 vs. 0.4 ± 0.97) and shared person in each party (2.04 ± 2.76 vs. 0.37 ± 0.80) before, compared to after MMT program, all differences were statistically significant (P < 0.001). We found that the average number of sexual acts per month with partners except spouse has decreased after MMT program (1.56 ± 3.36 vs. 1.11 ± 1.81, P = 0.008) while the average number of sexual acts with spouse per month increased from 0.85 ± 2.29 to 1.03 ± 2.24 (p = 0.007). In addition our results showed that the average numbers of unsafe sexual acts with opposite-sex partner per month before and after MMT program were 0.75 ± 1.54 vs. 0.51 ± 1.44 respectively (P < 0.001). Also after participation in MMT program the subjects reported lower numbers of unsafe homosexual acts per month (0.06 ± 0.38 vs. 0.2 ± 0.84, P < 0.001) (Table 2). 

**Table 2.** Risky Injecting and Sexual Behaviors Before and After MMT

Measure	Before MMT		After MMT		P value
	Mean	SD	Mean	SD	
**Number of injections per week**	21.29	15.11	7.74	7.58	< 0.001
**Number of sharing injections per week**	3.10	5.42	0.40	0.97	< 0.001
**Number of shared people in each party**	2.04	2.76	0.37	0.80	< 0.001
**Number of sexual contacts per month (except spouse)**	1.56	3.36	1.11	1.81	0.008
**Number of sexual contacts with spouse per month**	0.85	2.29	1.03	2.24	0.007
**Number of unsafe homosexual contacts per month**	0.20	0.84	0.06	0.38	0.001
**Number of unsafe heterosexual contacts per month**	0.75	1.54	0.51	1.44	< 0.001

Our results related to number of new infections incidence are summarized in Table 3. The estimated cumulative incidence of HCV per 100 IDUs due to sharing injection and unsafe sexual act before MMT program were 13.84 (95% CI: 6.17 - 21.51), 0.0003 (0.0001 - 0.0005) and after it were 36.48 (25.84 - 47.11) and 0.0004 (0.0002 - 0.0006) respectively. Based on our model total number of HCV infection averted per 100 IDUs per year was 22.64 (19.67 – 25.6). 

**Table 3.** Cumulative Incidence of HCV Infection per 100 IDUs per Year With /Without MMT

Outcome Measure	Without MMT			With MMT			Difference
	Mean	95% CI		Mean	95% CI		
		Low	high		Low	high	
**Sharing injection**	36.48	25.84	47.11	13.84	6.17	21.51	-22.64
**Sexual contact**	0.0004	0.0002	0.0006	0.0003	0.0001	0.0005	-0.0001
**Sum**	36.48	25.84	47.11	13.84	6.17	21.51	-22.64

**Figure 1.**
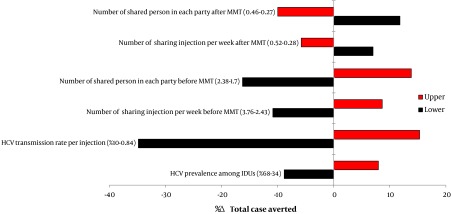
Results of One-Way Sensitivity Analysis (Tornado Diagram)

We also investigated how changes in model parameters would affect the total case averted using one-way sensitivity. Our results showed that changes in most of the input parameters had a few effects on the outcome. The total case averted were, especially, high sensitive to HCV transmission rate per injection, HCV prevalence among IDUs, number of shared person in each party and number of sharing injection per week (Figure 1). 

## 5. Discussion

Illicit injection drug use is an important public health problem around the world, recent global epidemiological data indicating that about 10 million IDUs have been infected with HCV (24). This study was conducted to examine effectiveness of the MMT program in preventing HCV incidence among IDUs. We found that the studied IDUs reported lower rate of risky injecting and sexual behavior after participation in MMT program. This finding is in agreement with other studies which examined the effectiveness of MMT and have shown reductions in risk behaviors, including needle sharing, number of sexual partners, engaging in sex without condom use, and exchange of sex for drugs or money (25-29).

The estimated cumulative HCV incidence shows that greatest part of the HCV incidence were due to injecting compared to sexual contact. It is partially explained by higher risk of transmission of HCV infection, in high risk injections, compared to unprotected sexual contacts (30-33), in addition, this study showed that the number of high risk sexual contacts in the IDUs was less than high risk injections.

We showed that for every 100 IDUs who participate in MMT program, 22.64 new HCV infections could be averted. In other words relative risk of HCV incidence in IDUs who are on MMT was about 38%. Craine and his colleague in a prospective cohort study showed that relative risk of HCV infection in IDUs who are in opiate substitution treatment was 34%. Protective effects of MMT on incidence of HCV infection have been reported in other studies too, ranged from 18% to 60% (34-37). However van Beek and his colleague found no association between opiate substitution treatment and HCV incidence among IDUs (38). In addition Crofts and his colleague reviewed effect of MMT on HCV infection incidence among IDUs from 1991 to 1995 and found that MMT is a risk factor for incidence of HCV infection (RR = 2.25) (39).

Nonetheless Hagan and his colleague have conducted Meta-analyse to estimate effects of risk-reduction interventions on HCV seroconversion and identify the most effective intervention types. Their results illustrated that MMT was one of the most effective interventions to prevent incidence of HCV among IDUs (estimated RR = 60%) (35). Although, most of literatures claimed that MMT can be effective, but there is a significant difference between estimated effectiveness of MMT. This difference could be originated from difference of modeling of calculating case averted, prevalence of HCV infection among IDUs population, and frequency of high risk behaviors among target groups.

Although using local high risk behavior data to estimate number of new cases of HCV among IDUs has made our results more applicable in local health policy making. Nonetheless our model has several limitations that merit consideration in interpreting results. First, our data were directly gathered from participants by an interviewer-administered questionnaire. Previous study has demonstrated that IDUs may under-report stigmatized behaviors, such as needle sharing and especially sexual behavior (40). Second, however local data sources were used wherever possible to ensure a high level of internal validity, but input data for some input parameters were derived from the international literature. These variables may therefore be different in Iranian patients. Nonetheless efforts were made to determine the sensitivity of our results to both structural and parameter uncertainty.

In conclusion MMT has been used for the treatment of addiction to heroin and other opiates for long time and has proven to be safe even when administered for 15 years or longer (41). IDUs not infected with HCV, who enter a MMT program and do not use other drugs or alcohol, are very likely to remain HCV-negative and given that many current and former MMT clients have sharing injections with other IDUs, therefore MMT is effective when broadly applied to a large fraction of active IDUs. Nevertheless, it is essential to compare its effectiveness with other interventions before implementing it in nationwide.
